# Lactoferrin gene knockdown leads to similar effects to iron chelation in human adipocytes

**DOI:** 10.1111/jcmm.12234

**Published:** 2014-02-26

**Authors:** José María Moreno-Navarrete, Francisco Ortega, Maria Moreno, Marta Serrano, Wifredo Ricart, José Manuel Fernández-Real

**Affiliations:** Service of Diabetes, Endocrinology and Nutrition, Institut d'Investigació Biomèdica de Girona (IdIBGi), CIBEROBN (CB06/03/010) and Instituto de Salud Carlos III (ISCIII)Girona, Spain

**Keywords:** lactoferrin, adipocytes, adipogenesis, iron metabolism

## Abstract

In human and mice adipose tissue, lactoferrin (LTF) has been found to be associated with increased adipogenesis and decreased inflammatory markers. Here, we aimed to investigate the effects of LTF knockdown (KD) in human adipocyte differentiation. In addition, the effects of exogenous LTF administration and iron chelation [using deferoxamine (DFO, 10 μM)] were tested. In both subcutaneous and visceral pre-adipocytes, LTF KD led to decrease significantly adipogenic, lipogenic and insulin signalling-related gene expression and a significant increase in the gene expression of inflammatory mediators. Human lactoferrin (hLf, 1 μM) administration led to recover adipocyte differentiation in LTF KD pre-adipocytes. Interestingly, iron chelation triggered similar effects to LTF KD, decreasing significantly adipogenic gene expressions. Of note, DFO (10 μM) and hLf (1 and 10 μM) co-administration led to a dose-dependent recovery of adipocyte differentiation. These new data reveal that endogenous LTF biosynthesis during human adipocyte differentiation is essential to achieve this process, possibly, modulating adipocyte iron homoeostasis. hLf administration might be a useful therapeutic target in obesity-associated adipose tissue dysfunction.

## Introduction

Lactoferrin (LTF) gene and protein expression has been detected at substantial levels in adipose tissue in direct association with adipogenesis and insulin signalling-related pathways and inversely associated with inflammatory markers [1]. In agreement with adipose tissue LTF gene expression, circulating LTF concentration was decreased in patients with type 2 diabetes [2], being its expression significantly reduced in neutrophils from these patients [3]. Interestingly, the main source of LTF in adipose tissue was the adipocyte fraction, being *LTF* mRNA and protein levels increased during adipocyte differentiation in a time course experiment [1]. Of note, in human pre-adipocytes exogenous human lactoferrin (hLf) administration led to increase adipocyte differentiation, enhancing PPARγ gene expression levels and insulin action, increasing insulin-induced ^pSer473^Akt and decreasing glucose concentration in the media [4]. This increase in insulin action is mediated by the increase in *GLUT4* and *IRS1* mRNA levels after exogenous hLf administration [1].

These previous findings led us to suggest that endogenous LTF levels in adipocytes might be involved in adipocyte differentiation. To confirm this hypothesis, in this study, we aimed to investigate the role of endogenous LTF biosynthesis in human subcutaneous and visceral adipocyte differentiation, performing *LTF* gene expression knockdown (KD) with short hairpin (sh)RNA lentiviral particles. In addition, the effects of exogenous LTF administration and iron chelation [using deferoxamine (DFO, 10 μM)] were tested.

## Materials and methods

### Differentiation of human subcutaneous and visceral pre-adipocytes

Isolated subcutaneous and visceral pre-adipocytes from human adipose tissue (Zen-Bio Inc., Research Triangle Park, NC, USA) from obese patients were plated on T-75 cell culture flasks and cultured and differentiated as previously reported [1, 4]. Briefly, isolated human subcutaneous and visceral pre-adipocytes were cultured (˜40,000cells/cm^2^) with pre-adipocytes medium (PM, Zen-Bio Inc.) composed of DMEM/Nutrient Mix F-12 medium (1:1, v/v), N-2-Hydroxyethylpiperazine-N′-2-ethanesulfonic Acid (HEPES), Fetal Bovine Serum (FBS), penicillin and streptomycin in a humidified 37°C incubator with 5% CO_2_. Twenty-four hours after plating, cells were checked for confluence (day 0^th^) and differentiation was induced using differentiation medium (DM, Zen-Bio Inc.) composed of PM, human insulin, dexamethasone (dxm), isobutylmethylxanthine (ibmx) and PPARγ agonists (rosiglitazone). After 7 days (day 7), DM was replaced with fresh adipocyte medium (AM, Zen-Bio Inc.) composed of DMEM/Nutrient Mix F-12 medium (1:1, v/v), HEPES, FBS, biotin, panthothenate, human insulin, dxm, penicillin, streptomycin and amphotericin. Negative control (non-differentiated cell) was performed with pre-adipocyte medium during all differentiation process. Fourteen days after the initiation of differentiation, cells appeared rounded with large lipid droplets apparent in the cytoplasm. Cells were then considered mature adipocytes, harvested and stored at −80°C for RNA extraction to study gene expression levels. The experiment was performed in triplicate for each sample.

### shRNA-mediated KD of LTF and hLf administration

Permanent silencing was performed with LTF-targeted and control (scrambled) shRNA lentiviral particles (sc-41371-V and sc-108080; Santa Cruz Biotechnology, Santa Cruz, CA, USA) and following the manufacturer instructions, during adipocyte differentiation. At day 14, cells harvested and stored at −80°C for RNA extraction and gene expression analysis after human adipocyte differentiation. hLf (Sigma-Aldrich, Barcelona, Spain; 1 μM) administration was performed with DM along 14 days in LTF-targeted shRNA cells. To test the effects of iron chelation, DFO (10 μM) alone and in co-administration with hLf (1 and 10 μM) were administrated during adipocyte differentiation in control shRNA cells.

### RNA expression

To study gene expressions, RNA was prepared from these samples using RNeasy Lipid Tissue Mini Kit (Qiagen, Izasa SA, Barcelona, Spain). The integrity of each RNA sample was checked by Agilent Bioanalyzer (Agilent Technologies, Palo Alto, CA, USA). Total RNA was quantified by means of spectrophotometer (GeneQuant, GE Health Care, Piscataway, NJ, USA) reverse transcribed to cDNA using High Capacity cDNA Archive Kit (Applied Biosystems Inc, Madrid, Spain) according to the manufacturer's protocol.

Gene expression was assessed by real-time PCR using a LightCycler® 480 Real-Time PCR System (Roche Diagnostics SL, Barcelona, Spain), using TaqMan^*®*^ technology suitable for relative genetic expression quantification.

The RT-PCR reaction was performed in a final volume of 12 μl. The cycle programme consisted of an initial denaturing of 10 min. at 95°C then 40 cycles of 15 sec. denaturizing phase at 95°C and 1 min. annealing and extension phase at 60°C. A threshold cycle (Ct value) was obtained for each amplification curve and a ΔCt value was first calculated by subtracting the Ct value for human *Cyclophilin A* (*PPIA*) RNA, which is used as housekeeping, from the Ct value for each sample. Fold changes compared with the endogenous control were then determined by calculating 2^−ΔCt^, so gene expression results are expressed as expression ratio relative to *PPIA* gene expression according to manufacturers' guidelines. The commercially available and pre-validated TaqMan® primer/probe sets used are provided at Data S1.

### Statistical analyses

Statistical analyses were performed with SPSS 12.0 software (SPSS, Chicago, IL, USA). Non-parametric tests, Mann–Whitney *U* and Wilcoxon's tests were used to evaluate the effects of LTF KD and hLf and DFO treatment. All these experiments were performed in triplicate.

## Results

### LTF KD effects on adipogenesis and inflammatory markers

**Figure 1:**
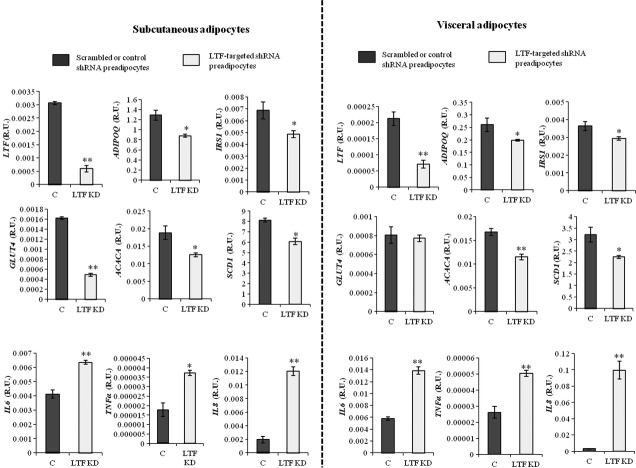
Effects of lactoferrin (LTF) knockdown (KD) on *LTF,* adipogenic (*ADIPOQ, ACACA, SCD1*), insulin-related (*IRS1, GLUT4*) and inflammatory (*IL6, TNFA, IL8*) gene expression during human subcutaneous and visceral adipocyte differentiation at day 14. *P < 0.05 in comparison with scrambled or control differentiated adipocytes. **P < 0.005 in comparison with scrambled or control differentiated adipocytes. Statistical analysis was performed with Mann–Whitney *U* and Wilcoxon's tests. These data are expressed as mean ± SEM of three independent experiments.

LTF KD resulted in consistently lower expression of LTF in both subcutaneous and visceral pre-adipocytes (80% and 65% respectively; Fig. 1) in parallel to decreased expression of adipogenic (*Adipoq, ACACA* and *SCD1*) and insulin-related genes (*GLUT4* and *IRS1*) in fully differentiated adipocytes (Fig. 1). On the other hand, the expression of inflammatory genes (*IL6, TNF*α and *IL8*) increased significantly in LTF KD adipocytes (Fig. 1), while LTF KD tended to decrease *LRP1* gene expression (Fig. S1A).

### Exogenous hLf effects on adipogenesis in LTF KD pre-adipocytes

**Figure 2:**
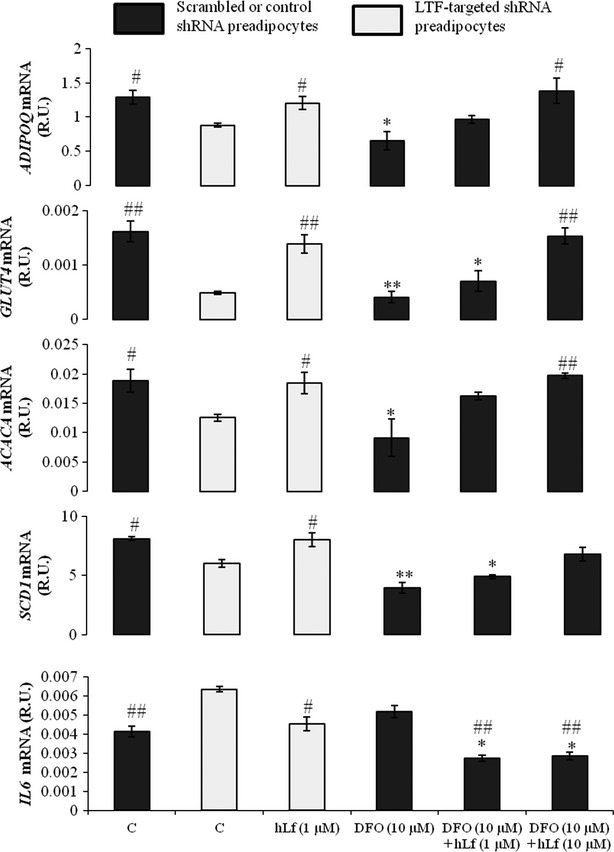
Effects of human lactoferrin (hLf; 1 μM) administration, deferoxamine (DFO) (10 μM) alone and DFO (10 μM) plus hLf (1 and 10 μM) co-administration on *ADIPOQ, GLUT4, ACACA, SCD1* and *IL6* on lactoferrin (LTF) knockdown (KD; grey bar) and scrambled or control (black bar) human pre-adipocytes during adipocyte differentiation at day 14. *P < 0.05 in comparison with scrambled or control differentiated adipocytes. **P < 0.005 in comparison with scrambled or control differentiated adipocytes. ^#^*P* < 0.05 in comparison with LTF KD differentiated adipocytes. ^##^*P* < 0.005 in comparison with LTF KD differentiated adipocytes. Statistical analysis was performed with Mann–Whitney *U* and Wilcoxon's tests. These data are expressed as mean ± SEM of three independent experiments.

Interestingly, hLf (1 μM) administration during adipocyte differentiation led to recover adipogenic gene expression and to reduce inflammatory gene expression in LTF KD fully differentiated adipocytes (Fig. 2 and Fig. S1B).

### Exogenous hLf effects on adipogenesis under iron chelation

Iron chelation in human subcutaneous pre-adipocytes showed similar effects that LTF KD, decreasing significantly adipogenic gene expressions (Fig. 2). Of note, DFO (10 μM) and hLf (1 and 10 μM) co-administration led to a dose-dependent recovery of adipocyte differentiation (Fig. 2).

## Discussion

To the best of our knowledge, this is the first study showing a functional role for endogenous LTF gene expression on human adipocyte differentiation, supporting previous data described in adipose tissue at the cellular level [1]. Lactoferrin gene expression in human adipose tissue was significantly decreased in obese patients and linked to the expression of several adipogenic and insulin-related genes, while negatively associated with inflammatory markers [1, 5–7]. Of note, the observed abnormalities in LTF KD cells were reversed after adding human exogenous LTF, increasing the expression of adipogenic genes while reducing inflammatory mRNAs. These results suggest that hLf administration might be a useful therapeutic target in those obese patients with adipose tissue dysfunction linked to decreased *LTF* mRNA levels.

Regarding the possible molecular mechanism for understanding the adipogenic effect of LTF, the well-known role of LTF in iron homoeostasis (its most ancestral function [8, 9]) might underlie the adipogenic effects of LTF. In fact, iron chelation disrupted human adipocyte differentiation in a similar manner to LTF KD, an effect that was reversed dose dependently by hLf co-administration. Recently, iron excess has been shown to induce iron accumulation in adipocytes leading to insulin resistance and decreased adiponectin production [10]. Both previous [10] and current findings suggest that disruption of iron homoeostasis (whether in excess or its deprivation) results in impaired adipocyte function (decreasing its adipogenic capacity). Thus, the adipogenic role of LTF might be mediated through the modulation of iron homoeostasis during adipocyte differentiation.

Furthermore, LTF KD tended to decrease *LRP1* gene expression (its putative receptor [11]) after adipocyte differentiation. A positive feedback of LTF on its receptor and itself was previously suggested [1], showing that exogenous hLf administration enhanced *LRP1* and *LTF* gene expression.

In summary, this study confirmed that the previously described autocrine production of LTF in adipose tissue [1] plays an essential role in human adipocyte differentiation, enhancing adipogenesis, insulin action and lipid replenishment in adipose tissue and reducing inflammatory mediators. The decrease in this protein in association with obesity could contribute to self-perpetuate insulin resistance-associated adipose tissue dysfunction [1] and might increase the negative effects of the iron homoeostasis disruption in adipose tissue. Here, we propose LTF administration as healthy therapeutic agent to improve obesity-associated adipose tissue dysfunction and its associated metabolic disturbances. Recent *in vivo* studies in humans and mice have shown that LTF administration exerts beneficial effects on obesity-associated metabolic disturbances, reducing visceral fat accumulation [12, 15]. However, further *in vivo* functional studies in humans are necessary to confirm hLf administration as potential therapeutic target.

## Supporting information

**Figure S1 (A)** Effects of LTF KD on LRP1 gene expression during human subcutaneous and visceral adipocyte differentiation at day 14. **(B)** Effects of hLf (1 lM) administration on IL8 and TNFa gene expression in LTF KD human preadipocytes *P > 0.05 in comparison with LTF KD differentiated adipocytes. **P > 0.005 in comparison with LTF KD differentiated adipocytes. Statistical analysis was performed using Mann Whitney U and Wilcoxon's tests. These data are expressed as mean ± SEM of three independent experiments.

**Data S1** The commercially available and pre-validated TaqMan® primer/probe sets used for gene expression analyses.
