# Supplementary figures and images for: Lactoferrin gene knockdown leads to similar effects to iron chelation in human adipocytes
Source: J Cell Mol Med. 2014 Feb 26;18(3):391–5. doi: 10.1111/jcmm.12234 (PMC3955146; doi:10.1111/jcmm.12234)

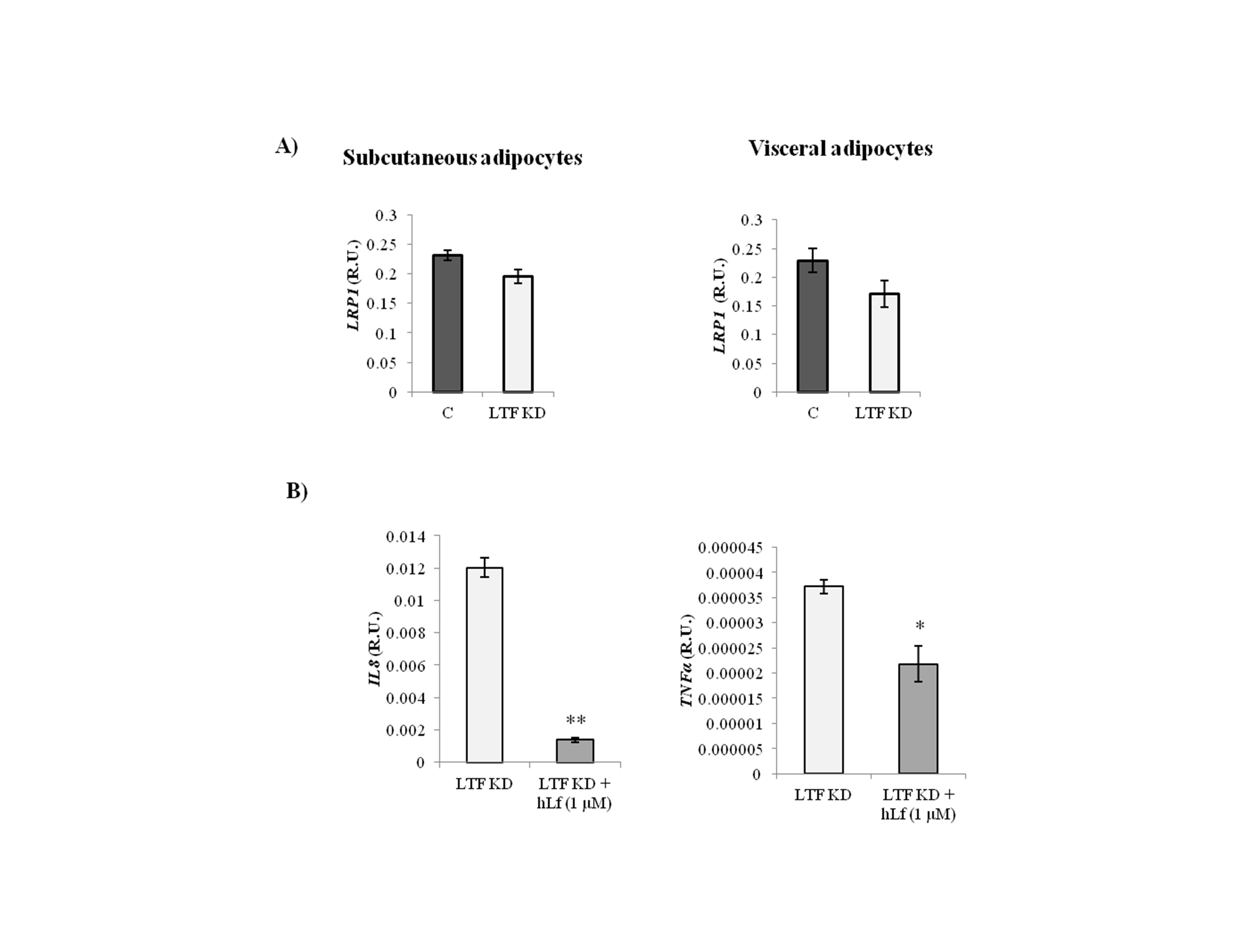

Supplement: Supplementary file 1 — Figure S1 (A) Effects of LTF KD on LRP1 gene expression during human subcutaneous and visceral adipocyte differentiation at day 14. (B) Effects of hLf (1 lM) administration on IL8 and TNFa gene expression in LTF KD human preadipocytes *P > 0.05 in comparison with LTF KD differentiated adipocytes. **P > 0.005 in comparison with LTF KD differentiated adipocytes. Statistical analysis was performed using Mann Whitney U and Wilcoxon's tests. These data are expressed as mean ± SEM of three independent experiments. [file jcmm0018-0391-sd1.tif]
