# Supplementary material for: Lactoferrin gene knockdown leads to similar effects to iron chelation in human adipocytes
Source: J Cell Mol Med. 2014 Feb 26;18(3):391–5. doi: 10.1111/jcmm.12234 (PMC3955146; doi:10.1111/jcmm.12234)
Supplement: Supplementary file 2 [file jcmm0018-0391-sd2.docx]

**Supporting information**

The commercially available and pre-validated TaqMan® primer/probe sets used for gene expression analyses

| Lactoferrin, **LTF** | Hs00914334_m1 |
| --- | --- |
| adiponectin, C1Q and collagen domain containing, **ADIPOQ** | Hs00605917_m1 |
| insulin receptor substrate 1, **IRS1** | [Hs00178563_m1](https://products.appliedbiosystems.com:443/ab/en/US/adirect/ab?cmd=ABAssayDetailDisplay&assayID=Hs00178563_m1&Fs=y&adv_phrase3=EXACT&adv_phrase2=EXACT&adv_phrase1=EXACT&assayType=GE&catID=601267&adv_kw_filter3=ALL&srchType=keyword&adv_kw_filter2=ALL&SearchRequest.Common.QueryText=irs1&kwdropdown=ge&adv_kw_filter1=ALL&species=Homo+sapiens&adv_query_text3=&searchType=keyword&adv_query_text2=&adv_query_text1=&uploadType=ID+List&adv_boolean3=AND&adv_boolean2=AND&adv_boolean1=AND&chkBatchQueryText=false&kwfilter=ALL&SearchRequest.Common.PageNumber=1&msgType=ABGEKeywordResults) |
| solute carrier family member 4, ***SLC2A4*** or ***GLUT4*** | Hs00168966_m1 |
| acetyl-Coenzyme A carboxylase alpha, **ACACA** | Hs00167385­_m1 |
| stearoyl-CoA desaturase (delta-9-desaturase)**, SCD1** | Hs01682761_m1 |
| tumor necrosis factor, **TNF** | Hs01113624_g1 |
| Interleukin 6, **IL6** | Hs00985639_m1 |
| Interleukin 8, **IL8** | Hs00174103_m1 |
| Low-density lipoprotein receptor-related protein 1, **LRP1** | Hs00233856_m1 |
| peptidylprolyl isomerase A (cyclophilin A)**, PPIA** | Hs99999904_m1 |
