# Supplementary material for: Lactoferrin gene knockdown leads to similar effects to iron chelation in human adipocytes
Source: J Cell Mol Med. 2014 Feb 26;18(3):391–5. doi: 10.1111/jcmm.12234 (PMC3955146; doi:10.1111/jcmm.12234)
Supplement: Supplementary file 3 — Data S1 The commercially available and pre-validated TaqMan® primer/probe sets used for gene expression analyses. [file jcmm0018-0391-sd3.docx]

**Supplementary online figure legends**

**Supplementary online figure 1.** **A)** Effects of *LTF* KD on *LRP1* gene expression during human subcutaneous and visceral adipocyte differentiation at day 14. **B)** Effects of hLf (1 μM) administration on *IL8* and *TNFα* gene expression in *LTF* KD human preadipocytes *p<0.05 in comparison with *LTF* KD differentiated adipocytes. **p<0.005 in comparison with *LTF* KD differentiated adipocytes. Statistical analysis was performed using Mann Whitney U and Wilcoxon’s tests. These data are expressed as mean ± SEM of three independent experiments.
